# Supplementary material for: Urinary interleukins (IL)-6 and IL-10 in schoolchildren from an area with low prevalence of Schistosoma haematobium infections in coastal Kenya
Source: PLOS Glob Public Health. 2023 Apr 5;3(4):e0001726. doi: 10.1371/journal.pgph.0001726 (PMC10075406; doi:10.1371/journal.pgph.0001726)
Supplement: Attachment — Submitted filename: Response to Reviewers.pdf [file pgph.0001726.s002.pdf]

Humphrey Kariuki Njaanake,
Department of Medical Microbiology &
Immunology,
P.O. Box 19676 – 00202,
NAIROBI – KENYA

10th May 2022

The Academic Editor
PLOS Global Public Health

Dear Sir,

RE: RESPONSE TO REVIEWERS' COMMENTS ON THE MANUSCRIPT: PGPH-D-22-00101 (URINARY INTERLEUKINS (IL)-6 AND IL-10 IN SCHOOLCHILDREN FROM AN AREA WITH LOW PREVALENCE OF UROGENITAL SCHISTOSOMIASIS IN COASTAL KENYA)

Thank you very much for taking your time to review our manuscript and giving very useful comments which have resulted in major improvement of the manuscript. The comments have been addressed in the manuscript as indicated below.

Journal Requirements/ Editor's Comments:

1. All trademark/copyright symbols have been removed.

2. The Introduction section has been included in the revised manuscript.

3. Figures 1 – 2 have been provided separately in the .tif format and all embedded figures removed from the manuscript.

4. The data set analyzed for this manuscript has been provided/ uploaded as Supplementary information.

5. The detailed Financial Disclosure statement has been amended and completed in full sentences.

6. The discussion has been reinforced and the conclusion rewritten.

The reviewers' specific comments have been addressed as follows:

Reviewer #1

Comment: The title is not concise and clear.

Response: The title has now been modified to read "Urinary interleukins (IL)-6 and IL-10 in schoolchildren from an area with low prevalence of *S. haematobium* infections in coastal Kenya"

Comment: The draft is composed of long sentences.

Response*:* This comment is not specific. The reviewer could have helped by suggesting which sentences are long and require shortening.

Comment: The references used in the study to justify the use of levels of cytokines and chemokine is far fetched since bacterial colonization in the urogenital organs and resultant pathology is different from parasitic infections.

Response: It is noteworthy that there is very little known about urinary cytokines in relation to schistosomiasis and the references used in the manuscript are to demonstrate that the urinary track tissues are immunologically active and can mount cytokine responses in inflamed state as would happen in urogenital schistosomiasis.

Comment: In *S. hematobium* infection, the cytokines released are as result of adult worms migrating into the venules surrounding the organs of the pelvis.

Response: This statement by the reviewer is not, at least partly, correct. In schistosomiasis, the adult worms live in the blood stream where they cause no tissue inflammation and thus no significant cytokine production by local tissues. Instead, it is the tissue-lodged eggs that provoke significant local inflammatory immune responses characterised by high amounts cytokines of tissue cell origin.

Comment: Therefore, the assay of IL-6 and IL-10 is an understatement. In this parasitic phenomenon we should assay for a panel of urinary cytokines and chemokines to weed out the most prominent ones.

Response: It is noteworthy that in one of the references (Njaanake et al., 2014) a number of cytokines were assayed and only IL-6 and IL-10 were positively and negatively correlated with urinary track pathology, respectively. This formed the basis of the present manuscript and made it prudent, as a follow up, to only assay the two relevant cytokines.

Comment: The author mixed up issues by further going looking at the effect of storage temperature on cytokine titers. This should have been an optimization protocol in the lab.

Response: The objective of this work was to assess factors that can potentially may make urinary cytokine (IL-6 and IL-10) levels a suitable tool to monitor urinary tract pathology due to *S. haematobium* infections. Storage temperature is one pertinent issue. Samples are usually collected in remote areas with no operational freezers and it was therefore important to assess how the levels of cytokines would be affected by specific temperatures above the usual refrigeration temperatures (i.e. $>^{-}20^{\circ}C$). This work was not intended to test the cytokine assays but to assess degradation of cytokines at different temperatures thus optimization protocol would have been inappropriate.

Comment: The author did not show any cytokine graphs or images of ultrasound detected pathologies.

Response: The graphs and figures have now been added as Fig 1, Fig 2 and Fig 3.

Reviewer #2

Comment: Title: " Urinary Interleukins (IL)-6 and IL-10 in schoolchildren from an area with Low Prevalence of Urogenital Schistosomiasis in coastal Kenya". Why the low prevalence region in coastal Kenya is the appropriate site of study?, please clarify or add this clarified info somewhere in the paper.

Response: This has now been clarified in page 4 of the manuscript. It was subsequent to a similar study carried out in hyperendemic area in coastal Kenya.

Abstract

Comment: "However, it is highly sensitive to urine storage temperatures", this sentence interrupts the flow of reading. Please exclude this, or add the result of temperature and IL-6 in the result section.

Response: This statement has been removed from the abstract.

Method

Comment: What is the inclusion /exclusion criteria of the participants?

Response: The inclusion/ exclusion criteria have been included in page 5. Only children aged between 5 and 15 years who were permanent residents of the area, have had no treatment with praziquantel in the past three months and had no observable clinical illness were randomly selected and included in the study.

Comment: Authors analyzed the effect of temperature on IL-6 in urine. How about the 2 consecutive urine samples were analyzed and interpreted.

Response: This could provide more insight into the IL-6 variation – It was assumed that immune responses due to tissue-lodged *S. haematobium* eggs that results in cytokine production has no daily variation unlike the parasite egg shedding. It is therefore prudent to analyse only one day urine sample for cytokines but, as recommended by WHO, three consecutive day urine samples for *S. haematobium* eggs.

Comment: How is the level of confidence to assess the level of hematuria from Dipsticks. Is this the limitation?

Response: Single use of dipsticks to detect haematuria in *S. haematobium* infections has been shown to be effective by several studies, some even recommending it as a proxy for infection with the parasites in children.

Comment: How the author confirms the result of *S. haematobium* eggs microscopic detection from the urine samples. Is this the limitation?

Response: *S. haematobium* eggs microscopic detection in urine was done as recommended by WHO and reported by several other published studies. This has even been used as a proxy for infection intensity and pathology. The method is however, not 100% sensitive although no any other better method is available.

Comment: Why not all samples were tested for ECP (n=164), IL-6 (n=165) and IL-10 (n=190).

Response: This has been explained in page 19 (Discussion). This was mainly due to breakage of vials.

Comment: Redundant info of ethical approval.

Response: This has been removed.

Result

Comment: Figure 1, how many times the author investigate the temperature effect to IL-6/IL10. I should be the error bar and statistical analysis.

Response: The effect of each specific temperature on urinary cytokines in each sample was investigated once but the samples, as recommended, were run in duplicates and an average calculated from the two readings. The graph (Fig 4) shows proportion of samples with detectable cytokines and it will therefore not be possible to add error bars on proportions unlike cytokine titres/ levels.

Discussion

Comment: Why aged group 10-11 has higher severe morbidity.

Response: This has been explained in page 16. This due to robust inflammatory responses early in pathology development but subsequently there is immunomodulation that reduces inflammation. Also, exposure to infections lessens with age.

Comment: Please add the rationale to focus only IL-10 and IL-6, why not other cytokines.

Response: This been added in pages 15 and 16. A previous study had shown the two cytokines to be the most relevant.

Comment: Why specificity of ECP, IL6 IL10 and haematuria are so low.

Response: This has been addressed in page 16.

Comment: The part of cytokines IL6 IL10 seems not relevant to the main objective. Two options 1. Keep it, but need to add some info to link this into the main text, this can make the study more beneficial to the readers 2. Exclude to make the study concise and focus.

Response: The part of ECP seemed ectopic and misplaced in the study. To make the study concise and focused, this part has been removed.

Comment: Conclusion can be rewritten. Some info should be in the discussion rather than the conclusion.

Response: This has been rewritten and extraneous material moved to discussion.

Minor comment

Comment: Title of table 2 should be revised, it is not only the sensitivity.

Response: This has been changed to include specificity.

Reviewer #3

Comment 1: The title is not appropriate with the objective of the study. The study has shown the relationship with the IL-6 and IL-10 with the detection of *S. haematobium* egg. In that circumstances, the study will be design as diagnostic validity of IL-6 ad IL-10. Please explain.

Response: Whereas the study has shown the relationship between cytokines (IL-6 and IL-10) and *S. haematobium* eggs in urine, it is not the only relationship shown. The relationships between the cytokines and other variables (e.g. haematuria and ultrasound-detectable pathology) have also been shown and therefore limiting the title to design to diagnostic validity will be inappropriate and misleading.

Comment 2: The methodology section of abstract is missing with the study design, study place, study period. It should be mentioned in this section.

Response: The study design, study area and period have been mentioned in the Abstract section.

Comment 3: The introdction is nicely written. but the rationale of the study is missing.

Response: The study rationale has been added to the introduction section.

Comment *4:* The methodology section is nicely written. The details of the ELISA procedure can be avoided. No need to elaborate the procedure. Please mention "Th ELISA was performed accoridng to the manufacturer's instruction."

Response: Details of the ELISA procedures have been removed and the statements “ELISA was performed according to the manufacturer’s instructions” added.

Comment *5:* The results section should be written according to the objective of the study. The relevant table should be mentioned.

Response: The results sections has been rewritten in line with the main objective of the study.

Comment 6: Rewrite the conclusion related with the findings of the study.

Response: The conclusion has been rewritten in line with the findings of the study.

I hope that our manuscript will now be considered favourably.

Once again thank you for offering us a chance to improve the manuscript for publication.

Kind regards

Yours faithfully,

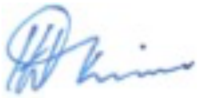

Humphrey Kariuki Njaanake
