# Supplementary material for: Urinary interleukins (IL)-6 and IL-10 in schoolchildren from an area with low prevalence of Schistosoma haematobium infections in coastal Kenya
Source: PLOS Glob Public Health. 2023 Apr 5;3(4):e0001726. doi: 10.1371/journal.pgph.0001726 (PMC10075406; doi:10.1371/journal.pgph.0001726)
Supplement: Attachment — Submitted filename: Rebuttal Letter II.pdf [file pgph.0001726.s003.pdf]

PGPH-D-22-00101
Urinary Interleukins (IL)-6 and IL-10 in Schoolchildren from an Area with low Prevalence of Urogenital Schistosomiasis in coastal Kenya
PLOS Global Public Health

Dear Sir,

RE: RESPONSE TO REVIEWERS' COMMENTS ON THE MANUSCRIPT: PGPH-D-22-00101 (URINARY INTERLEUKINS (IL)-6 AND IL-10 IN SCHOOLCHILDREN FROM AN AREA WITH LOW PREVALENCE OF UROGENITAL SCHISTOSOMIASIS IN COASTAL KENYA)

Thank you very much for taking your time to review our manuscript and giving very useful comments which have resulted in major improvement of the manuscript. The comments have been addressed in the manuscript as indicated below.

MS ID: PGPH-D-22-00101R1 Title: Urinary Interleukins (IL)-6 and IL-10 in Schoolchildren from an Area with low Prevalence of Schistosoma haematobium infections in coastal Kenya

REVIEWER #5

GENERAL

This manuscript investigated the association between urinary cytokine levels (IL6 & IL10) with the prevalence, intensity and morbidity of *S. haematobium* infection among schoolchildren in Kenya.

I've been given this manuscript as a revised (R1) version and I noticed that the manuscript has been improved after addressing reviewers' comments given during previous reviewing stage. However, the manuscript still has major drawbacks in quality of writing and data & results presentation. Major and minor comments are provided below. They should be addressed before this manuscript can be acceptable for publication.

MAJOR REVISION

1. In methods section, Study area and study design, page 6: It is stated that "A third part of day-1 urine sample was divided into three aliquots which were stored at –20 C, 4 C and 25 C, respectively, for 14 days before being assayed for IL-6 and IL-10." That's said, all three portions of 1st sample were kept for 14 days; so, why ILs were not assessed directly after sample collection? How ILs can be considered as markers if examined after 14 days?
   Response: *The urine samples were stored at different temperatures for 14 days before cytokine assaying in order to demonstrate the effect of storage temperatures outside freezer. This is explained in the last paragraph of the introduction as part of the objective. Assaying all the samples on day I, as the reviewer is requiring, would mean pursuing a different objective and thus carrying out a different study.*

2. Discrepancies in data and results were noticed and the results might be considered unsafe. For instance,

2.1. Table 3: "61 were infected" but they were 60 in table 2 (36+24). Please revise.
Response: *This was a typographical error and has since been corrected on Table 3.*

2.2. Table 3: 29 children had haematuria, i.e. 17.6% but it is stated that "the prevalence of haematuria among the children was 14.8%."
Response: *It has been shown clearly that Table 3 presents data from 165 children for whom urine samples were assayed for IL-6 and not the initial 245. Therefore if 29 children out of the considered 165 children had haematuria, this simply translates to 17.6% (i.e., 29/165) and not 14.8% (i.e., 29/245) as the reviewer has stated.*

2.3. Table 3: distribution of children age groups is also different from table #2: 54, 88 and 23 in table 3 while appear as 40, 89 and 36 in Table 2. A big difference! Thus, results should be revised in table and text, including statistics.
Response: *The distribution of the children's age groups in Table 3 is different from the distribution of children's age distribution in Table 2. Table 2 has not presented age distribution. However, the age distributions in Tables 1 and 3 have been harmonized and the statistics were not affected at all.*

2.4. Table 4: numbers of children in the groups (sex, age, infection, haematuria) are different from tables 2 and 3. This is strange. The total of children here in table 3 is not 165; e.g. 189 (106 girls + 83 boys)! And 190 (72 positive + 118 negative)!! The results are considered unsafe.
Response: *This comment is not clear. The tables (2 and 3) have different number of children. This is because Table 2 does not give the number of children anywhere. Whereas urine samples from 165 children were assayed for IL-6, urine samples from 190 children were assayed for IL-10 (Table 4) for the reason given as a limitation (page 15). Therefore, numbers of children (n) in the two tables cannot be the same. The typographical error (n=106) in Table 3 has been corrected to n=107.*

2.5. Table 2: this is incomplete. Provide the raw data in terms of numbers of positive and negative results of each test compared to microscopy then these results of sensitivity and specificity can be shown. Moreover, add results of PPV, NPV & Kappa statistics. This table was based on 165 children.
Response: *This has now been done.*

2. Many other comments on results reporting are provided below, refer to minor comments.

.

3. The manuscript needs stylistic and English editing to improve the quality of writing should be improved. For example:
Response: *This has now been done.*

3.1. Long sentences in all sections including abstract should be split into short and clearer sentences.

Response: Long sentences have shortened.

3.2. Some grammatical and typographic errors were noticed.

Response: Where these errors have been noticed, the same have been corrected in the revised version.

3.3. Some incomplete sentences; e.g. last 2nd line to end in page 18 "pathology in the urinary tracts of the was comparable to", and some other sentences in different sections.

Response: The sentence on page 18 had a typographical omission and has since been corrected. Others, where noticed, have also been corrected.

3.4. Text, particularly results section can be largely shortened.

Response: This has now been extensively done.

3.5. Methods, Text of haematuria: "Briefly, Avoiding touching the ………. side of the testing strip container." Remove all these unnecessary details

Response: These have been removed.

3.6. Discussion section should also be rewritten. It should be more focused and concise.

Response: This has now been. The Discussion have been rewritten and focused. It has been made more concise.

3. 7. Avoid over repetition of results in discussion section.

Response: This has been corrected.

4. What is the study design? It is stated in abstract that it was a cross-sectional study but not mentioned in "Study area and study design". This should be stated with more information in methods section. Moreover, it seems to be a cross-sectional controlled study, taking in account the 165 children only, 60 (cases) were infected and 105 were not infected (controls). However, description on how those 165 were selected was not described. What was the rationale of selecting the school? How children were selected at the school? Similarly, the area was described as low prevalence area in the title but this was not mentioned or described in methods.

Response: *The correction has been made in page 5. However, the no controlling was consciously done in the study design but, rather, only comparisons of groups were done. In addition, not all children availed themselves for all the tests. Those who did not avail some specimens or data were not systematically different from the others and therefore it is no appropriate to only consider 165 children. That the area, compared to "Njaanake* et al.*, 2014", was a low endemic area has been mentioned in the methodology.*

5. In general, the manuscript can be entirely rewritten based on 165 children selected for assessing the association between ILs and infection. Describe the main survey first with 425 children then 165 were selected (explain), and focus on the 165 children. Minimize text about demographic factors associated with infection. For example,

Discussion, 3rd paragraph "Boys had higher prevalence …": This paragraph can be shortened or ignored. The core of this study was the 165 children, and the epidemiological results might be incomplete as some important factors were not assessed. When stating "suggest possible gender-based differences in exposure with boys having a higher exposure." This needs other factors to be investigated and confounders to be controlled. Refer to major comments.

Response: *There were 245 children and not 425 children as stated by the reviewer. Whereas 165 children had their urinary tracts examined for morbidity and their urine specimens assayed for IL-6, 190 children had their urine specimens assayed for IL-10. It therefore follows that restricting the study to 165 children then data from 25 children will be lost. It was not possible for all the children to undergo all the tests as these were not done at one point or in one day. It is noteworthy that the statement that "suggest possible gender-based differences in exposure with boys having a higher exposure" is a suggestion, based on well known facts in schistosomiasis, and not a conclusion. This statement has been removed.*

MINOR REVISION (arranged according to text flow)

6. Abstract, 1st sentence: remove "due to infections with *Schistosoma haematobium"*.

Response: *This was removed.*

7. Abstract, 2nd sentence: The sentence can be removed. "However, there is no universally recommended tool for assessing the inflammation and resultant morbidity especially in early stages."

   Response: *This was removed.*

8. Abstract, methods: split the 1st sentence into short sentences.

Response: *The first sentence in the Abstract is already short and shortening it further will make it lose its meaning.*

9. Abstract, results: last sentence is unclear; split this long sentence into short sentences.

   Response: *This was corrected.*

10. Abstract, Conclusion: positively! How this was concluded?

    Response: *This has now been rephrased.*

11. Abstract, Conclusion: "prevalence of urinary IL-6 is positively associated with *S. haematobium* infection and morbidity". Morbidity!. But it was stated that "*S. haematobium*-related urinary tract morbidity using ultrasonography" & "There was no significant association between prevalence or levels of IL-6 and ultrasound detectable".

    Response: *This has now been corrected.*

12. Introduction, last sentence: "…to assess how urinary IL-6 and IL-10 are associated to age and sex in children ….". This is slightly different from the general objective

mentioned in the abstract. Please rephrase following general objective that focuses first on the association with urinary tract schistosomiasis. You may change “to assess how ….. associated with ..” to “to assess the association of …. With …”.
Response: *This has now been done.*

METHODS:

13. “which is endemic for *S. haematobium*.” This is referred to the school not the area! Please rephrase.
    Response: *This was corrected.*
14. “infested with *S. haematobium* snail intermediate hosts,” cite a reference to support this statement.
    Response: *This has been removed.*

15. “Urine samples from the 2 subsequent days were only examined for presence of *S. haematobium* eggs..” If eggs were detected in the 1st sample, why other 2 samples examined? If to get better epg estimation, please cite related reference.
    Response: *This has been corrected.*

16. Urine examination for *S. haematobium* eggs: “One urine sample (10 ml) was collected from each child on each of three days and” this repetition can be removed.
    Response: *This has been corrected.*

17. Urine examination for *S. haematobium* eggs: “or heavy if □50 eggs or more” the symbol is enough or rephrase.
    Response: *This has been corrected.*

18. Urinary tract ultrasound examination: “Ultrasound examination of the urinary tracts of each child was performed” This statement is not in line with results and table 1 that showed only 165 children underwent this examination. This should be stated here and justified or explained.
    Response: *This has been corrected.*

19. Statistical Analysis: “Medians of *S. haematobium* eggs, IL-6 and IL-10 were compared using Mann-Whitney U tests or Kruskal-Wallis test, as appropriate.”
    Response: *This has now been modified.*

20. Methods: study population should be described in this section. Information on the targeted population, selection, sample size estimation should be provided.
    Response: *This has now been done.*

RESULTS:

21. Study Population: “Urine samples from the children were examined for *S. haematobium* eggs.” Remove this statement.

Response: *This has now been done.*

22. "The overall prevalence ….. was 36.3%. The ……..was 50.5% for boys and 25.2% for girls" the proportions should be accompanied with the numbers; i.e. was 36.3% (89/245). Is 89 the true number of infected children? Please check. Apply this where applicable.
Response: *This has now been done.*
23. "…, with higher odds of infection than female (p<0.001, odds ratio (OR) =0.32, 95% CI: 0.18 – 0.56). This statement is not compatible with the statistics (OR & 95%CI) that showed a lower odds of infection among females compared to males. Report exactly what statistics reveal.
Response: *This has now been done.*

24. "Boys had significantly higher *S. haematobium* egg counts than girls (p<0.001)." Please state the values (mean or medians or geometric mean) for each group and provide the statistical test computed value/coefficient before the p value.
Response: *Based on the distribution of eggs, this statement is not very informative and has been removed.*
25. "(X2= 11.538; p = 0.003)." why Chi square was reported here while OR & 95%CI were reported for sex.
Response: *This has now been corrected by only using* X^2.
26. "had significantly higher egg counts". Please state the values (mean or medians or geometric mean) for each group.
Response: *Based on the distribution of eggs, this statement is not very informative and has been removed.*
27. "A total of 165 children had their urinary tracts examined for morbidity using ultrasound." How was these 165 children selected? When (before or after urine examination)? How many of them infected? 60 infected and 105 not as shown in table 1; this should also be stated and explained in the text.
Response: *This has been corrected.*
28. "The intensity of morbidity ranged from none to severe (Fig 1)." Fig. 1 does not show this range of morbidity!
Response: *This has been corrected.*

29. "A significantly higher proportion of boys had ultrasound-detectable morbidity than girls (X2= 18.0760; p<0.001)." This should be revised and rephrased. Higher proportion of girls had light morbidity than males, although the overall proportion of the boys was higher.
Response: *This has been corrected.*
30. "Significantly higher proportion of children with infection had pathology (X2= 14.3969; p<0.002)" This should be revised and rephrased. Which group had higher proportions at morbidity groups? 29.2% of heavily infected children had moderate morbidity, this was over double the proportion of light infected and over 4 times of not infected, etc.

Response: *This has been corrected. The term overall has been inserted in the general statement to avoid repeating all the information already presented in the table.*

31. "no significant difference in proportions of children with pathology in relation to age (X2= 10.2826; *p*=0.113)." This also should be revised and rephrased. As seen, 9% of those aged 10 – 11 years had severe morbidity compared to 0 and 2% of others. And 30.5% of 12-15 years had light morbidity …. etc.
Response: *This has been corrected. The term overall has been inserted in the general statement to avoid repeating all the information already presented in the table.*

32. Haematuria: "Boys had significantly higher odds of having haematuria than girls with prevalences of 21.9% and 9.4%, respectively (*p*<0.008, OR =0.37, 95% CI: 0.18 – 0.77). This statement is not compatible with the statistics (OR & 95%CI) that showed a lower odds of among females compared to males. Report exactly what statistics reveal.
Response: This has been rephrased.
33. Haematuria: "positive association" what does positive mean? Does it mean, increase as the age increased? Explain or rephrase.

Response: *The term positive association is a commonly used term in statistics and it means when considering two variables, when one is high the second variable also tends to be high as opposed to negative association which means that when one variable is high the second tends to be low (https://www.statisticshowto.com/direction-of-association/). The term positive association was therefore used appropriately to reduce the amount of text.*

34. "… were haematuric." Please rephrase.

Response: *This has been rephrased to "had haematuria"..*

35. "60.0% of children with heavy" please add the numbers beside the proportions. Apply to all results section and abstract.
Response: *This has been effected.*

36. "The sensitivity and specificity of IL-6 and IL-10 ELISA and haematuria were calculated using microscopy detection of *S. haematobium* eggs as the gold standard (Table 2)." Please rephrase to improve clarity. The results were not calculated by microscopy, but they were compared and microscopy was the reference group, etc.
Response: This has been corrected.

37. IL-6 in relation to sex and age: "Urine samples from 165 children were assayed for IL6 levels." This is not mentioned in methods.
Response: This has been corrected.

38. Results: Text is too lengthy. Sections IL-6 and IL-10 in relation to morbidity, Effects of Temperature on IL-6, and Effects of Temperature on IL- 10 can be largely shortened and merged where applicable.

Response: This has been addressed (Shortened).

39. Citing the figures in text should be revised. E.g. Fig. 3 was cited in section IL-10 in relation to sex and age but not in IL-6 and IL-10 in relation to morbidity. Too confusing.

Response: This has been addressed.

40. Figures 2 and 3 can be improved. Otherwise add column for the mean or median levels of the IL in the relevant tables and label with significant statistics or add another column for p values for levels difference.
Response: A column for medians and a column for their respective p-values have been added in Tables 3 and 4 for IL-6 and IL-10, respectively.

DISCUSSION:

41. Discussion, 1st paragraph: text can be shortened.
Response: This has been addressed.
42. Discussion, 2nd paragraph "Haematuria": many studies have evaluated the performance of haematuria. Thus, related discussion should be improved.
Response: This has been addressed.
43. Discussion, 3rd paragraph "Boys had higher prevalence …": This paragraph can be shortened or ignored. The core of this study was the 165 children, and the epidemiological results might be incomplete as some important factors were not assessed. When stating "suggest possible gender-based differences in exposure with boys having a higher exposure." This needs other factors to be investigated and confounders to be controlled. Refer to major comments.
Response: This has been addressed.

44. Reference list should be prepared following journal's style. E.g. do not capitalize all words of journal articles titles, but journal names should be capitalized, etc.
Response: This has been corrected.

REVIEWER #6

The research manuscript is focused on the development of new diagnostic tools for the estimation of the prevalence and intensity of Schistosomiasis haematobium in sub-Saharan Africa. This is a critical area of study considering the global need for effective and efficient diagnostics for schistosomiasis in the drive towards the control of this disease and the need to eventually transition to the elimination of the disease. The need for appropriate diagnostics is one that should be clearly emphasized in the manuscript to further drum home the importance of the paper and its findings.
The objectives of the research are well justified. Many schistosomiasis programs in SSA are challenged with the need for appropriate diagnostic tools for undertaking schistosomiasis

parasitological assessments. In my opinion, the methods section is adequate and meets the ethical requirements of the study. The analysis is rigorous and adequate to support the results, discussions, conclusions, and recommendations of the study. It establishes the association between schistosomiasis morbidity and IL-6 and IL-10 by clearly indicating a strong association with IL-6 and but no significant association with IL-10. The paper provides relevant evidence in support of the research agenda to develop diagnostics for schistosomiasis control and elimination programs.
As indicated earlier the statistical analysis, in my opinion, is suitable and appropriate for the study. The results obtained help to establish the associations between schistosomiasis morbidity and IL-6 and IL-10. The parameters studied, that is age and sex of the study population and storage temperatures of the urine samples that led to the establishment of a positive association between IL-6 and schistosomiasis morbidity are quite rigorous.
The paper is well-written and presented in clear and easy-to-understand language. Though certain portions are repetitive, they otherwise help in clarifying the findings of the study. There are a few typographical errors that will hopefully be corrected by the editorial team. The manuscript meets the publication criteria and I recommend it for publication after ensuring improvements in the editorial quality and flow of the paper.
Response: *These comments mostly overlap with comments by Reviewer #5 and have therefore been addressed concurrently.*

We wish to thank you for offering us a chance to revise the manuscript for publication.

Kind regards

Yours faithfully,

Humphrey Kariuki Njaanake
